# Student and the Lanarkshire milk experiment

**DOI:** 10.1007/s10654-022-00941-x

**Published:** 2022-12-07

**Authors:** Stephen Senn

**Affiliations:** School of Health and Related Research, University of Sheffield, Sheffield, UK

**Keywords:** Cluster design, Incomplete blocks, Random effects, Standard errors, Nutrition, Randomisation, Student

## Abstract

A detailed examination of the 1930 Lanarkshire Milk Experiment (LME) by the famous statistician William Sealy Gossett (“Student”), which appeared in *Biometrika* in 1931, is re-examined from a more modern perspective. The LME had a complicated design whereby 67 schools in Lanarkshire were allocated to receive either raw or pasteurised milk but pupils within the schools were allocated to either receive milk or to act as controls. Student’s criticisms are considered in detail and examined in terms of subsequent developments on the design and analysis of experiments, in particular as regards appropriate estimation of standard errors of treatment estimates when an incomplete blocks structure has been used. An analogy with a more modern trial in osteoarthritis is made. Suggestions are made as to how analysis might proceed if the original data were available. Some lessons for observational studies in epidemiology are drawn and it is speculated that hidden clustering structures might be an explanation as to why results may vary from observational study to observational study by more than conventionally calculated standard errors might suggest.

*It need hardly be said that to carry out an experiment of this magnitude successfully requires organisation of no mean order and the whole business of distribution of milk and of measurement of growth reflects great credit on all those concerned.* Student [1] (P398)

## Background

The chronology of the publication by William Sealy Gossett (1876–1937) ‘Student’ of a commentary of the Lanarkshire Milk Experiment (LME) is carefully described in chapter five of Egon Pearson’s biography [2] of Student, which is the chapter devoted to Student’s scientific exchanges with RA Fisher. (See in particular pp 60–65.)

In 1930, Leighton and McKinley had written a report [3] describing a large dietary experiment on schoolchildren in Lanarkshire. Some 20,000 children in all were to be studied, the subjects being recruited from 67 schools. The plan was to give five thousand *feeders* raw milk, five thousand *feeders* pasteurised milk with ten thousand *non-feeders* acting as controls. (In the end rather fewer than planned children provided data.) The duration of the experiment was four months and the growth of the children in terms of weight and height assessed. For practical reasons, the type of milk that was given was varied between but not within schools, each school providing feeders and non-feeders but none permitting a direct comparison of raw and pasteurised milk. Furthermore allocation of type of milk to school was not randomized although some attempt at randomisation within schools between feeders and non-feeders was made. Thus, the experiment is of a form that we might now describe as a *cluster allocated incomplete block design*.

As the introduction to the report explained [3], various experiments in the USA and the UK had ‘demonstrated the high nutritive value of milk as a supplementary ration in children,’ (p 2) but they were open to various criticisms which the new study by virtues of its size and the care with which it was conducted was meant to avoid. However, criticism was not avoided. The report concluded that the effects on weight and height of pasteurised or raw milk were similar. but this claim was soon challenged [4] by the agricultural scientist Stephen Bartlett and a follow up note in April of 1931 with Fisher [5] claimed that Leighton and McKinlay’s conclusions was, ‘open to some question’ (p 591). This note seems to have attracted Student’s attention, since in a letter to Karl Pearson (KP) of 14 July 1931, he mentioned that he was currently examining the official report and also Fisher and Bartlett’s note. On the 23 of July he sent KP a draft of his analysis of the data. KP replied with detailed comments on 26 July to which Student sent an equally detailed reply on 30 July. The corrected proofs of his piece were sent back by Student on 18 August and were published in Pearson’s journal *Biometrika* in the December issue of that year. One can only marvel at the speed with which our scientific forbears managed these things in an era before electronic communication.

### The report

In order to understand what Student was able to do and what he could not do in reanalysing the data, it is necessary to know the nature of the summary statistics available to him from the report by Leighton and McKinlay.

The original raw data consisted of initial and final weights (in lbs) and heights (in inches) of the pupils. Leighton and McKinlay summarised these data in 12 Tables. Each Table had 7 rows, one for each age group from 5 to 11. The number of columns varied but a major grouping gathered the data in two sections, one for Boys and one for Girls. Thus in total there were $7 \times 2 = 14$ age-by-sex groups. A summary of the the tables provided by them is given in Table 1 as follows.

**Table 1:** Summary of tables in the LME report

Table	Description of statistics tabulated	Additional information
1	Average initial weight by group	Numbers of pupils
2	Average initial heights by group	
3	Differences in average initial weights by group between treatments	Probable errors
4	Differences in average initial heights by group between treatments	Probable errors
5	Correlations between original values and change for controls for (1) weight and (2) height	Probable errors
6	Average increase in weights in the three groups	
7	Average increase in heights in the three groups	
8	Differences of changes in weights between two milk groups and controls	Probable errors
9	Differences of changes in heights between two milk groups and controls	Probable errors
10	Correlations between original values and change for raw milk for (1) weight and (2) height	Probable errors
11	Correlations between original values and change for pasteurised milk for (1) weight and (2) height	Probable errors
12	Differences in changes of between raw and pasteurised milk for weight and height	Probable errors

A major deficiency in reporting is that data were not tabulated by school. In fact, the report does not even make clear in how many schools pupils were given raw milk and in how many pasteurised. Various commentators, including Student, assumed for argument’s sake that the split might have been 33, 34, which, given the total of 67, is as close to equal as can be managed. The total numbers tabulated for the three treatments are given in Table 2. In total 4375 + 5221 = 9596 pupils were given milk and of these 45.6% received raw milk. If this percentage is applied to 67, the total number of schools, it gives 31 schools to the nearest whole school, which would imply that there were 36 schools given pasteurised milk. Of course the numbers per school were not equal, so that could explain the discrepancy (five fewer schools rather than just one). Not so easily explained is the discrepancy between numbers of non-feeders and feeders. The difference of 978 amounts to nearly 15 pupils per school, which seems rather large, given that the average number enrolled was 272. Also strange is that Leighton and McKinley refer to 17, 159 records as being useable (p12) rather than the 18,214 suggested by their table.

**Table 2:** Numbers of pupils by sex and treatment in the LME

Treatment	Total control	Raw	Pasteurised	Total
*Sex*				
Boys	4320	2236	2088	8644
Girls	4298	2139	3133	9570
Total	8618	4375	5221	18,214

Based on Table 2 of the report

No information is given as to how the probable errors were calculated. But a footnote to Table 5 states:

The figure after the ± sign in this and other tables is the probable error, which affords a measure of the reliability of the result. A difference or a coefficient of correlation equal to or greater than three times this figure is generally regarded as significant. At the same time, in any series of results, such as is given in Table 5, great importance must not be attached to isolated “significant” coefficients. Attention should rather be given to the run of the results.(P15)

The probable error of a statistic is its semi-interquartile range. For a Normal distribution this is approximately 2/3 of the standard error, so that three probable errors are about two standard errors. Thus the ‘significance’ standard being proposed here is similar to the modern 5% which would correspond to about two standard errors. It is not clear, however, how Leighton and McKinley obtained the probable errors and it is doubtful that they would have been able to calculate them in a way that took account of the hierarchical nature of the data (pupils within schools). The advice to look at the run of the results, is wise.

For further discussion of the LME and other related experiments of the era, the reader is referred to Pollock’s interesting paper [6].

### Student’s arguments

Student makes a number of perceptive points about the study, many of which are still relevant generally as regards observational studies and clinical trials. I shall make some links to modern concerns in the next section but will limit myself in this section to outlining Student’s main points under the following headings: (1) Basic design, (2) Selection of pupils, (3) Measurement of weights and (4) Pooling of controls.

#### Basic design

Student notes and accepts that for practical reasons it was necessary to use only one type of milk in a given school. Nevertheless, he points out that this has an unfortunate effect on precision, since no direct comparison between raw and pasteurised milk can be made within schools. As he puts it: ‘this does introduce the possibility that the raw and pasteurized milks were tested on groups of children which were not strictly comparable’ (P398). Nowadays, we would deal with this in one of two ways: either by treating school as a fixed factor with 67 levels or by nominating school as a random main effect factor. The latter would be more efficient but would be vulnerable to bias if allocation of milk type to school was not random. See “Appendix” for a discussion of the precision of these two approaches.

#### Selection of pupils

Student notes that allocation of selected pupils either to receive milk or to act as controls was made either by ballot (that is to say randomly) or using an alphabetical system. However, he notes that this form of allocations was sometimes ‘improved’ by adjusting the allocation to make comparison fairer, or as the report put it, ‘In any particular school where there was any group to which these methods had given an undue proportion of well fed or ill-nourished children other were substituted…’ [3] (P7). He then writes [1]: ‘In this case it was a fatal mistake, for in consequence the controls were, as pointed out in the Report, definitely superior both in weight and height to the “feeders” by an amount equivalent to about 3 months’ growth in weight and 4 months’ growth in height.’ (p 399).

**Fig. 1:**
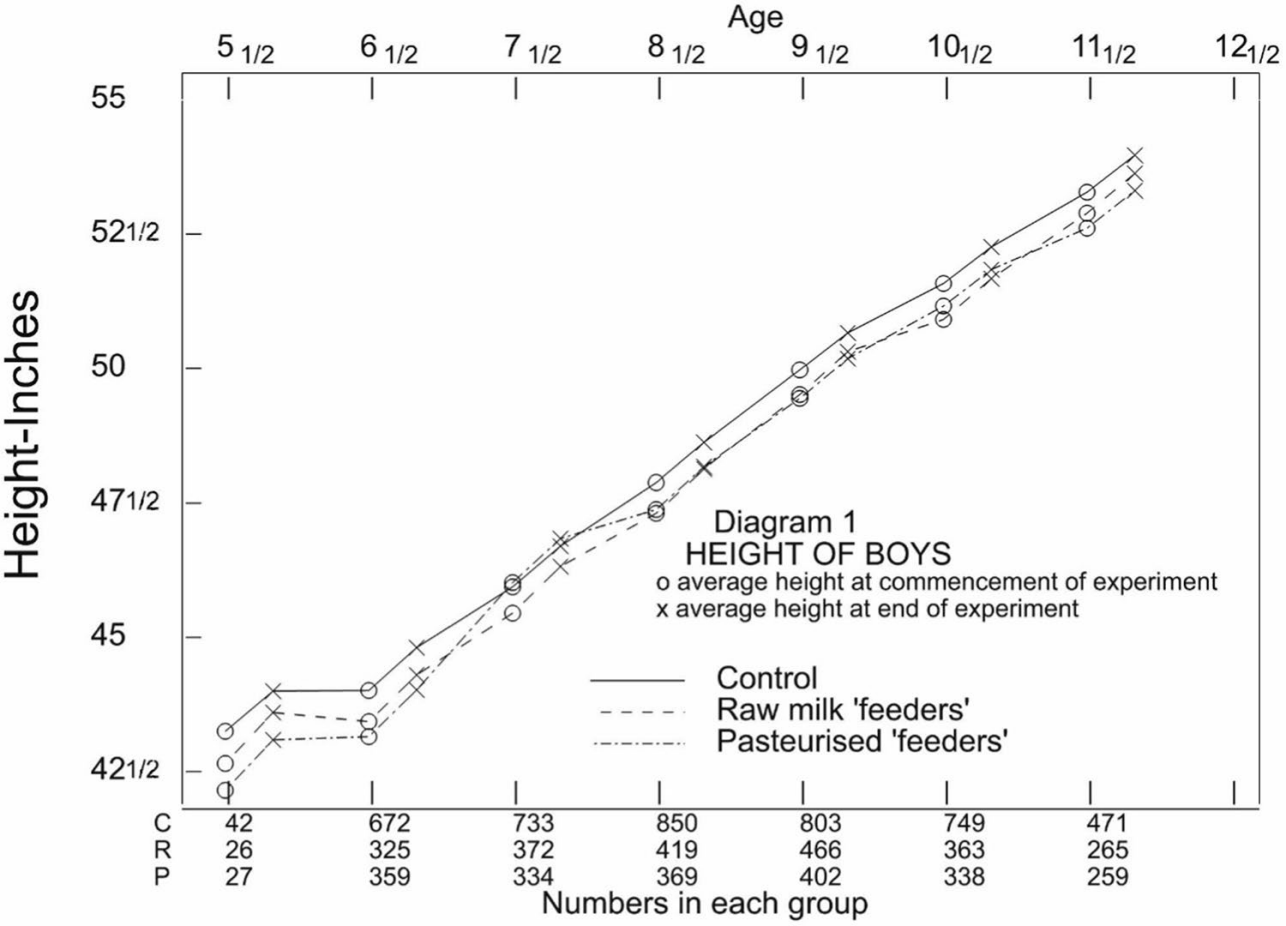
Reproduction of Diagram 1 from Student’s paper. Height by age cohort for boys enrolled on the LME. Note that a baseline height is indicated o and that at 4 months by x. Pairs of o followed by x are measured in the same age cohort. However, although all points are joined, in moving from x to o a different age cohort is introduced

Final heights and weights are not tabulated in the LME report but the differences to baseline are. Student calculated these final figures and plotted them against age separately for boys and girls together with the baseline values. Figure 1 is an attempt to provide a faithful representation of Diagram 1 in Student’s paper. He plots heights for all 7 age cohorts at baseline (labelled o) and at outcome (labelled x). Note that he joins all these points together but that involves age varying not only over joining pairs of points within cohorts but also between cohorts. The points labelled o at any age should only reflect differences due to allocation. It is noticeable that the control value is the highest for six out seven cohorts. His Diagram 2, which shows heights for girls, shows that the control value is the highest for all seven cohorts. In other words, the baseline differences do not appear to be random.

Later in his commentary Student remarks:

To sum up: The Lanarkshire experiment devised to find out the value of giving a regular supply of milk to children, though planned on the grand scale, organised in a thoroughly business-like manner and carried through with the devoted assistance of a large team of teachers, nurses and doctors, failed to produce a valid estimate of the advantage of giving milk to children and of the difference between raw and pasteurized milk.

This was due to an attempt to improve on a random selection of the controls which in fact selected as controls children who were on the average taller and heavier than those who were given milk.

The hypothesis is advanced that this was due not to a selection of the shorter, lighter children as such to take the milk, but to an unconscious bias leading the teachers to pick out for this purpose the needier children whom the milk would be most likely to benefit. (p 406)

From a modern perspective, either lack of or failure with a randomisation process is an obvious flaw. It is perhaps worth noting, however, that when it came to agricultural experiments, with which he was more familiar, Student favoured systematic arrangements over random ones such as had been proposed by Fisher and this led to a public disagreement between them [7, 8]. See ‘Added values’ [9] for a discussion.

#### Measurement of weights

**Fig. 2:**
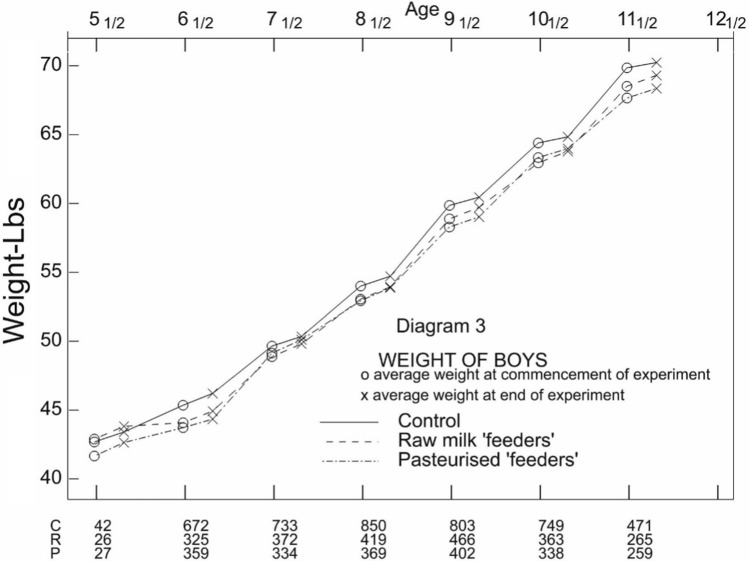
Reproduction of Diagram 3 from Student’s paper. Weight by age cohort for boys enrolled on the LME. Note that a baseline weight is indicated o and that at 4 months by x. Pairs o followed by x are measured in the same age cohort. However, although all points are joined, in moving from x to o a different age cohort is introduced

From his Diagram 3 (weight for boys reproduced as Fig. 2 here) and his Diagram 4 (weight for girls, not reproduced here) Student notes:

Here there is, after the first two ages, a very decided dip, especially in the later ages. The weights at the end of the experiment are too low. This might be accounted for by a tendency in older children to grow normally in height and subnormally in weight during the spring, but I think it much more likely that older children weigh about 1 lb. more clothes in February than they do in June, while in the case of younger children a more limited wardrobe permits of fewer discards. (P 172)

This remark underlines the value of two things. First, that causality in a controlled experiment should not be judged by comparing outcome with baseline but comparing the experimental group(s) with control: other things being equal this phenomenon would affect feeders and non-feeders alike. Secondly, the value of concurrent control.

#### Pooling of controls

A serious error, which has affected subsequent attempts to interpret the data, including this one, was that control data were summarised in terms of a single control group. As Student points out:

Now with only 67 schools, at best 33 against 34, in a district so heterogeneous both racially and socially, it is quite possible that there was a difference between the averages of the pupils at 33 schools and those of pupils at another 34 schools both in the original measurements and in the rate of growth during the experiment.

In that case the average “control” could not be used appropriately to compare with either the ”raw” group or the “pasteurized” group. (p170).

This will be discussed in the next section and is also investigated in the “Appendix”.

### A more modern perspective

A more modern study using the same sort of design as the LME is the TARGET trial [10–12]. This study in osteoarthritis enrolled about 18, 244 subjects, so a very similar number to the LME. In total 849 centres were involved and for practical reasons in some centres subjects were randomised to either Lumiracoxib or Naproxen and in others to either Lumiracoxib or Ibuprofen [12]. Thus the trial, just like the LME, is a cluster allocated incomplete blocks design, with centres analogous to schools, Lumiracoxib analogous to no milk and Ibuprofen and Naproxen analogous to raw and pasteurised milk (or vice versa). A detailed discussion of design aspects of this trial has been given elsewhere [13].

Both experiments for practical reasons thus consist of two sub-studies, there being no random allocation of centres to sub-study but a strict randomisation within centres per sub-study in TARGET and a partially effective attempt at something similar in the LME.

Various outcomes were assessed in TARGET but one that is analogous to weight and height in the LME, in that it is continuous and measured at baseline and at outcome, is blood pressure, about which it is stated, ‘Analysis of blood pressure data used ANCOVA on average blood pressure changes across all post-baseline assessments, with baseline values and sub-study as covariates.’[10] (P 678). The TARGET study and its analysis thus provides a useful perspective on what Leighton and McKinley and subsequently Student did with the LME.

According to William Cochran [14], the rudiments of Analysis of Covariance (ANCOVA) were introduced by RA Fisher in the 4th edition [15] of *Statistical Methods for Research Workers* (SMRW) in 1932 and he had completed the theory by the 5th edition [16] of 1934. In fact, there was an earlier paper by Bailey [17] dating from 1931 which describes the approach as being a natural extension of ideas of Fisher’s and Student’s and, indeed, the first edition of SMRW [18] discusses sampling errors of regression coefficients, the theory being provided in a paper of Fisher’s [19] of the same year, 1925.

Be that as it may, Leighton and McKinley cannot really be blamed for not using the baselines as a covariate in their analysis, since the techniques was scarcely used, if at all, by the time of their report. Instead, they chose to analyse the change scores (difference between final and initial values) for weight and height. They calculated correlation coefficients between baselines and change -scores and found these to be modest, which implies that the correlation between baselines and outcomes was high and therefore that the analysis of change scores was nearly fully efficient.

However, their decision to pool the two control groups was not logical and even if, as is discussed in the “Appendix”, schools had been randomly allocated to receive raw or pasteurised milk, would almost certainly have increased the variance of the overall estimate compared to just using the local control. Since schools were not randomly allocated, the argument applies *a fortiori*: they were introducing a bias into what might have been an unbiased estimate. In the TARGET study, for example, the authors dealt with this by fitting sub-study as a covariate. This deals effectively with the bias problem since it forces the contrasts of interest to be with the relevant control group. The only issue raised is that the variance will be estimated from all three treatments, even when only comparing two.

**Fig. 3:**
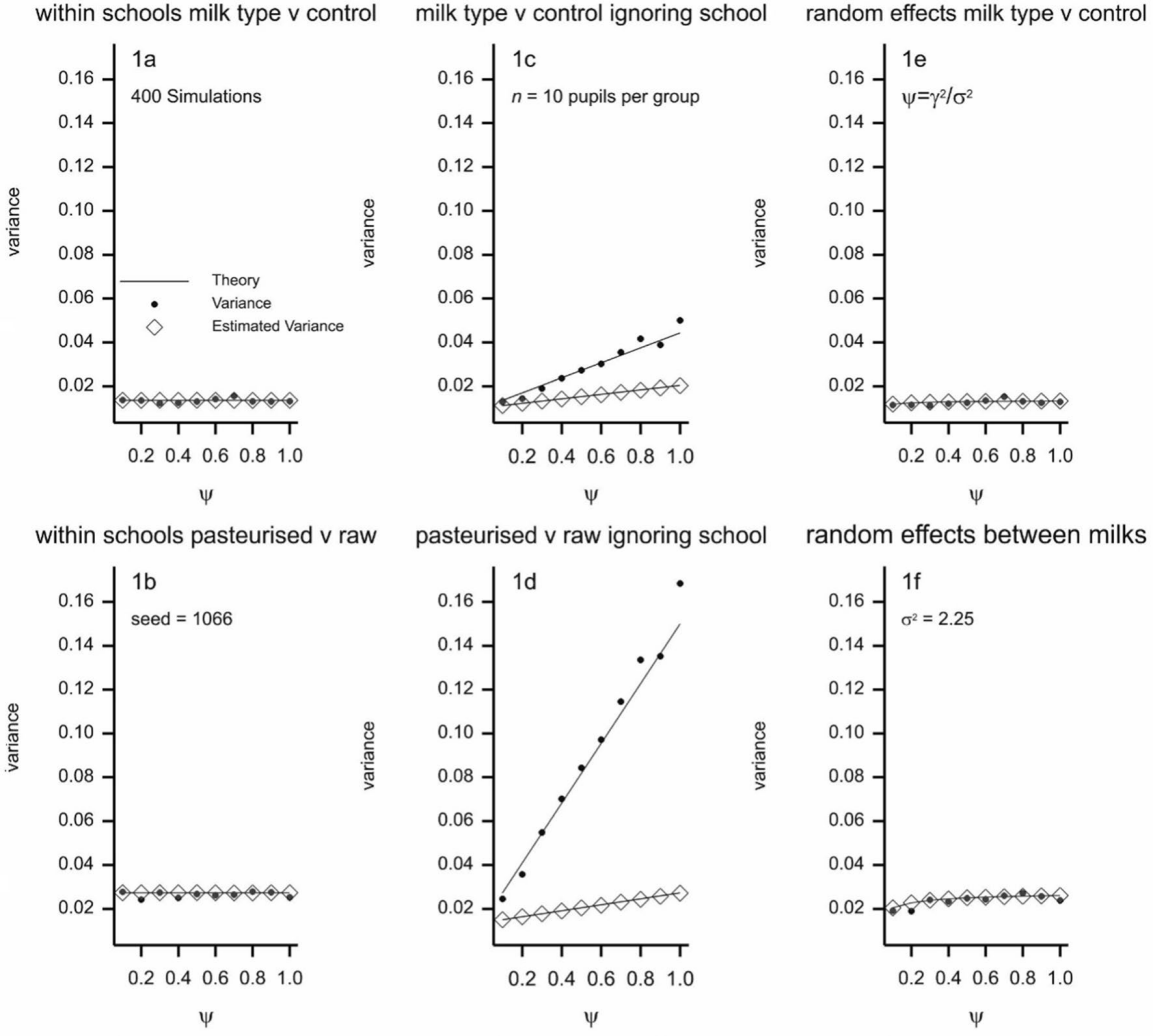
Variances and estimated variances for two types of contrasts and three different approaches to analysis. Top row: milk type versus control. Bottom row: comparison of milk types. Left hand column: analysis treating school as fixed. Right hand column: analysis treating school as random. Middle column: incorrect analysis ignoring school effects. Lines show theoretical values as given in the “Appendix”, points show simulated variances and diamonds show simulated estimated variances. A group represents one of the 28 age by sex by milk received combinations. It is assumed that will be 10 pupils per school for such a group and, for simplicity 33 school of each type. The within group variance is assumed to be 2.25 inches squared. The ratio of the between to within school variance is given by $\psi$. The results of 400 simulations are shown

To compare raw milk and pasteurised milk, Leighton and McKinley should have used the method of a double contrast. At the time this was not mainstream practice, however, and it was work [20, 21] later in the decade by Fisher’s assistant and successor at Rothamsted, Frank Yates, that established the appropriate analysis of incomplete block designs. A lot of this work has been reinvented (not always as well) in the last few decades in connection with network meta-analysis. The possible consequences of various types of analysis strategy are illustrated in Fig. 3 where the two central panels show that naïve pooling and not allowing for school effects would lead to a considerable inflation of variances and an underestimate of that inflation. Thus estimated standard errors would be incorrect. (See “Appendix” for explanation.)

Pupils within schools are an example of a hierarchical block structure. Analysis of experiments that have hierarchical structures can be confusing and difficult and, indeed, as Cochran points out [14], Fisher got an early example [22] wrong. (See also Yates’s commentary [23] of 1964.) However, By 1930 Fisher had achieved a deep understanding of the various issues raised by such experiments and had developed the tools to deal with them. That being so, his piece with Bartlett is not particularly impressive but then he did not have access to the original data and had to rely on the summary statistics provided by Leighton and McKinley.

### How should the data be analysed?

Here I offer my perspective under seven headings; Sex, Sub-study effects, School effects, Baselines, Age, Other covariates, Multivariate analysis. First, however one important point is that any serious analysis would require use of the original data. In fact, Dr Ethel Elderton obtained the original cards, which had been lent to Karl Pearson by the Department of Health for Scotland and published an analysis in 1933 in *The Annals of Eugenics *[24], no doubt as a result of much laborious transcription. Unfortunately, she appears to have made no use of the fact that the data were clustered in schools: not even to the extent of resolving the mystery of how many there were of each type. George Davey Smith informs me that the cards were still available in 1988. The notes below are my recommendation for what an appropriate analysis might be for anybody with the energy and time to process them.

#### Sex

Given the large number of pupils involved, in my opinion there is not much to be gained by a joint analysis of boys and girls. This would impose similar covariances and variances for the two sexes and my preferences would be a separate analysis for each with a possible comparison of results afterwards to see if there was a treatment-by-sex interaction.

#### Sub-study effects

Given that allocation of raw or pasteurized milk to schools was not at random, it essential that sub-study should be included as a fixed effect factor with two levels. This is what was done in the TARGET study.

#### School effects

Given that sub-study will be fitted as an effect, there will be no inter-school information of the sort discussed in the “Appendix” to be recovered when comparing raw and pasteurised milk. However, if the numbers of feeders and non-feeders are not balanced school by school, there will be a small amount of information recoverable by treating school as random when comparing (say) raw-milk feeders with raw-milk controls. My feeling is that this is not really worth it, the information gain would be small and there would be some risk of bias, and that therefore school should be fitted as a fixed effect factor with 37 levels. Since schools are nested within sub-study, if that is the case it is immaterial whether sub-study is included as a factor or not.

#### Baselines

Baselines should be fitted as a covariate. This would make a valuable contribution to reducing variability of the measurements and whether final values or change scores are used, the result will be the same [25]. There are two issues, however. First, if school has been fitted as a random effect (ignoring advice above), then there are two covariances, not only within but between schools, and dealing with this can be a delicate matter [26]. Second, it might be of interest to examine the treatment-by-baseline interaction. My advice would be that this should be a secondary question to be investigated.

#### Age

Given the design, age is not a biasing factor but it is possible that fitting age as a further factor in a model would make a contribution to reducing variability. My preference would be to fit it as a continuous variable, rather than as a grouped categorical variable, a popular but not necessarily logical habit, that is both arbitrary and order invariant. Of course the effect of age is already partly captured by using baseline values but there may be further useful information. Age as a single linear predictor would go a long way to capturing further variation. Splines [27], fractional [28] or orthogonal [29] polynomials are alternatives.

#### Other covariates

In principle there is no reason why baseline height should not be used as a covariate when analysing weight and vice versa.

#### Multivariate analysis

There could be some interest in studying the joint distribution of final height and weight as a function of treatment.

## Conclusion

The LME is of considerable historical interest, in particular because it attracted the attention of some of the statistical giants of the day, including Fisher, Karl Pearson and Student, whose commentary has been the particular focus here. The points he made are perceptive and still valuable. Whether the LME itself is of any value in informing our understanding of nutrition is doubtful. For one thing, even if the original data were processed and even if an appropriate analysis was employed, it is doubtful that adjusting for the measured covariates would deal with the bias in allocation within schools. By the standards of modern randomised trials the study is inadequate. It is not clear, to me, however, that the data would be inferior to what one might expect from the sort of observational study that is frequently used to draw epidemiological conclusions. As the “Appendix” shows, it is likely that the authors will have underestimated the true variance of the contrasts they used. It is possible that many observational studies suffer (unwittingly) from the same defect, in that they may contain hidden clusters that will inflate the true standard errors but not the estimated ones. A recent review by Cox, Kartsonaki and Keogh [30] had this to say.

Most although not all relatively standard statistical procedures produce, after due precaution against anomalies, estimates with standard errors inversely proportional to the square root of sample size. For big data these standard errors are thus likely to be extremely small…In fact there is evidence from many fields that when data are examined with a broad horizon standard errors may decrease inversely as a smaller power of sample size, for example as the one-quarter power. (P 114)

Collignon et al. [31] made a similar point

…observational studies… are conventionally analysed as if they were less-than-perfect parallel group trials with adjustment for ‘confounders’ being the solution for dealing with the imperfection. That is to say, compared to a clinical trial, a penalty is paid for the loss of orthogonality that confounding brings, but otherwise the variance term is treated as if a parallel group trial were appropriate. Many cohort studies are analysed exactly like this. In other words, the problems we have described raise the following possibility, namely that confounding is not the only problem with observational studies. A further problem is the implicit assumption of conditional independence of observations given adjustment. (P1969)

Of course, there may also have been much work since on the effect of milk on growth of children that would make its conclusions irrelevant.

## Appendix: Variances of contrasts from a cluster allocated incomplete block design

Assume for simplicity that we have two treatments $T_{1} ,T_{2}$ each of which is compared to a control $C$ within $k$ clusters, each of which has $n$ subjects allocated to treatments and $n$ allocated to control. In practice, of course, designs will not be so well-balanced, and neither the LME nor the TARGET study were, but this simple unrealistic set-up is sufficient to make some useful points. Assume that the between-cluster variance is $\gamma^{2}$ and the within-cluster variance is $\sigma^{2}$. (In the discussion that follows it will be implicitly assumed that the ratio of ${{\gamma^{2} } \mathord{\left/ {\vphantom {{\gamma^{2} } {\sigma^{2} }}} \right. \kern-\nulldelimiterspace} {\sigma^{2} }}$ is known. In practice it has to be estimated and the estimate is a random variable. This makes the formulae that follow approximate only [32].) Where the control appears in the same clusters as $T_{1}$ we can refer to it as $C_{1}$. A simple estimate of the effect of $T_{1}$ compared to $C$ is to use the within-cluster contrast, which, using a rather loose notation, can be labelled, $T_{1} - C_{1}$ (where it is to be understood that this is formed using the relevant means in the clusters in which both are represented). If that is so, the between-cluster variance is irrelevant and the contrast will have variance

1 $$\frac{{\sigma^{2} }}{kn} + \frac{{\sigma^{2} }}{kn} = 2\frac{{\sigma^{2} }}{kn}$$

Obviously, the same variance applies to the contrast $T_{2} - C_{2}$.

The cluster effects can be eliminated from the contrast $T_{1} - T_{2}$ by taking the double difference $\left( {T_{1} - C_{1} } \right) - \left( {T_{2} - C_{2} } \right)$, since $C_{1} ,C_{2}$ are assumed to have the same effect in expectation. The two halves of this double contrast are independent so that the estimate will have a variance equal to the sum of the two and thus be double that of the treatments with their control. The resulting variance will be

2 $$2\frac{{\sigma^{2} }}{kn} + 2\frac{{\sigma^{2} }}{kn} = 4\frac{{\sigma^{2} }}{kn}.$$

A refinement might be to also use the between-clusters information. This analysis proceeds by noting that $\left( {T_{1} + C_{1} } \right)$ is independent of $\left( {T_{1} - C_{1} } \right)$ and $\left( {T_{2} + C_{2} } \right)$ is independent of $\left( {T_{2} - C_{2} } \right)$. Thus $\left( {T_{1} + C_{1} } \right) - \left( {T_{2} + C_{2} } \right)$ is a further independent estimate of the differences in the effects of the two treatments. This, however, is a between-clusters estimators and will have variance

3 $$\left( {\frac{{4\gamma^{2} }}{k} + \frac{{2\sigma^{2} }}{kn}} \right) + \left( {\frac{{4\gamma^{2} }}{k} + \frac{{2\sigma^{2} }}{kn}} \right) = \frac{{8\gamma^{2} }}{k} + \frac{{4\sigma^{2} }}{kn}$$

The optimal estimator will be a weighted combination of the within-cluster and between-cluster estimates with weights proportional to the reciprocals of their respective variances. If $w$ is the weight for the within-clusters estimate so that $1 - w$ is the weight for the between-clusters estimate we have,

4 $$w = \frac{{\sigma^{2} + 2n\gamma^{2} }}{{2\left( {\sigma^{2} + n\gamma^{2} } \right)}},\quad 1 - w = \frac{{\sigma^{2} }}{{2\left( {\sigma^{2} + n\gamma^{2} } \right)}}.$$

The resulting variance is

5 $$\left( {\frac{{2\sigma^{2} }}{kn}} \right)\left( {\frac{{\sigma^{2} + 2n\gamma^{2} }}{{\sigma^{2} + n\gamma^{2} }}} \right).$$

If there is no additional between-cluster variability (that is to say none above that due to the random variation between subjects), $$\gamma^{2} = 0$$ and then $$w = {\raise0.5ex\hbox{$\scriptstyle 1$} \kern-0.1em/\kern-0.15em \lower0.25ex\hbox{$\scriptstyle 2$}}$$ in (4) and with and between estimates are weighted equally. Where this is the case, the second term in (5) is 1 and hence (5) is the same as (1). On the other hand as $${{n\gamma^{2} } \mathord{\left/ {\vphantom {{n\gamma^{2} } {\sigma^{2} }}} \right. \kern-\nulldelimiterspace} {\sigma^{2} }} \to \infty$$, then $$w \to 1$$ and the second term in (5) approaches 2 and so the value of (5) approaches that of (2), reflecting the fact that the variance of (3) is so high that no between-clusters information is recoverable.

At one time, this approach to recovering what was referred to in the experimental design literature as ‘inter-block information’ was popular for computational reasons [21]. Nowadays an approach using a mixed model with the main effect of school as random might be more usual [33].

However, if, as seems likely, Leighton and McKinley simply compared $T_{1}$ and $T_{2}$ directly, the variance would have a contribution from the cluster effects and would be

6 $$2\left( {\frac{{\gamma^{2} }}{k} + \frac{{\sigma^{2} }}{kn}} \right).$$

That being so, it seems unlikely that they will have estimated the probable errors correctly. Furthermore, if they compared $T_{1}$ (say) to the simple unweighted mean of $C_{1}$ and $C_{2}$, the resulting estimate would then have a variance of

7 $$\frac{{\gamma^{2} }}{2k} + \frac{{3\sigma^{2} }}{2kn}.$$

The condition that (7) is less than (1) is that

8 $$\frac{{\gamma^{2} }}{{\sigma^{2} }} < \frac{1}{n}.$$

Note that since the mean number of pupils studied per school was about 270, implying $n \simeq 135$, the ratio of between to within variances would have to be very low to satisfy this. The consequence is that it would usually be better to ignore the controls from the other schools when comparing (say) $T_{1}$ to $C$.

Note that naïve estimates of the variances given by (6) and (7) would be

9 $$\frac{{2\left( {\gamma^{2} + \sigma^{2} } \right)}}{nk}$$

and

10 $$\frac{{3\left( {\gamma^{2} + \sigma^{2} } \right)}}{2nk}.$$

By inspection, it is clear that the estimated and true variances will only be identical if $\gamma^{2} = 0$ and as is shown by the middle panels 1c and 1d of Fig. 3, the discrepancy increases markedly as the ratio of the between and within school variances, $\psi = {{\gamma^{2} } \mathord{\left/ {\vphantom {{\gamma^{2} } {\sigma^{2} }}} \right. \kern-\nulldelimiterspace} {\sigma^{2} }}$ increases.
